# Association between Non-HDL-C/HDL-C Ratio and Carotid Intima–Media Thickness in Post-Menopausal Women

**DOI:** 10.3390/jcm11010078

**Published:** 2021-12-24

**Authors:** Arcangelo Iannuzzi, Francesco Giallauria, Marco Gentile, Paolo Rubba, Giuseppe Covetti, Alessandro Bresciani, Emilio Aliberti, Gilanluigi Cuomo, Camilla Panico, Maria Tripaldella, Maria Ausilia Giusti, Alessandro Mattina, Gabriella Iannuzzo

**Affiliations:** 1 Department of Medicine and Medical Specialties, A. Cardarelli Hospital, 80131 Naples, Italy; lelliann@libero.it (A.I.); giuseppe.covetti@aocardarelli.it (G.C.); alessandro.bresciani@aocardarelli.it (A.B.); 2 Department of Translational Medical Sciences, Federico II University of Naples, 80131 Naples, Italy; francesco.giallauria@unina.it (F.G.); gianluigi.cuomo95@gmail.com (G.C.); 3 Department of Clinical Medicine and Surgery, Federico II University, 80131 Naples, Italy; margenti@unina.it (M.G.); rubba@unina.it (P.R.); mariatripaldella@gmail.com (M.T.); 4 Department of Gastroenterology, North Tees University Hospital, Stockton-on-Tees TS19 8PE, UK; ealiberti@hotmail.co.uk; 5 Diagnostic and Interventional Radiology-Policlinico Gemelli Foundation, 00168 Rome, Italy; camillapanico33@gmail.com; 6 Diabetes and Islet Transplantation Unit, Department of Diagnostic and Therapeutic Services, IRCCS-ISMETT (Istituto Mediterraneo per I Trapianti e Terapie ad alta specializzazione), UPMC Italy, 90127 Palermo, Italy; giustiausilia@gmail.com (M.A.G.); alessandromattina@gmail.com (A.M.)

**Keywords:** non-HDL-C/HDL-C ratio, carotid intima-media thichness, post-menopausal women

## Abstract

Atherogenic lipoproteins (particularly, very low-density lipoproteins, VLDL) are associated with subclinical atherosclerosis. The present study aims at evaluating whether routinely analysed lipid parameters are associated with carotid intima–media thickness, a proxy for subclinical atherosclerosis. Lipid parameters from 220 post-menopausal women undergoing ultrasound investigation of the carotid arteries were analysed. Forty-five percent of women showed subclinical atherosclerosis on carotid ultrasound. The mean carotid intima–media thickness was 1.26 ± 0.38 mm. The mean value of the non-HDL-C/HDL-C ratio was 3.1 ± 1.2. Univariate analysis showed a significant association between non-HDL-C/HDL-C ratio and intima–media thickness (r = 0.21, *p* = 0.001). After adjusting for cardiovascular risk factors (age, systolic blood pressure, smoking, body mass index Homeostasis model assessment: insulin resistance and high-sensitivity C-Reactive-Protein), multivariate analysis showed a significant association between non-HDL-C/HDL-C ratio and intima–media thickness (β = 0.039, *p* = 0.04). Logistic regression analysis showed that the highest tertile of the non-HDL-C/HDL-C ratio was associated with the presence of carotid plaques (OR = 3.47, *p* = 0.003). Finally, a strong correlation between non-HDL-C/HDL-C ratio and cholesterol bound to VLDL (r = 0.77, *p* < 0.001) has been found. Non-HDL-C/HDL-C ratio is associated with the presence of carotid atherosclerosis in post-menopausal women and is strongly correlated to VLDL-C levels.

## 1. Introduction

After menopause, women lose the characteristic protection from cardiovascular events from which they benefit in their fertile years, probably due to the action of oestrogens [1]. It is well known that low-density lipoproteins (LDL) are the main determinant of atherosclerosis and of its clinical sequelae [2]. However, there are other cardiovascular risk factors, which promote atherosclerosis even when LDL levels are normal or when they are controlled pharmacologically, so it has been coined ‘residual risk’ [3]. Notably, although metabolic syndrome strongly impacts cardiovascular risk [4,5] LDL-C levels are not included among its definition criteria. In Southern Italy, there is a high prevalence of obese women, who often present with metabolic syndrome, with insulin resistance and a prevalence of cardiovascular disease comparable to males of the same age. In the “Progetto CUORE”, which looked at the association between risk factors and incidence of cardiovascular events, a significant relationship between metabolic syndrome and 10-year cardiovascular risk was found, which was stronger in women than in men (hazard ratios of 1.74 and 1.34, respectively) [6]. It should be noted that not only LDL but also triglyceride rich lipoproteins (TRL), which contain apo-B and have a diameter <70 µm, are able to pass through the endothelial barrier and tend to accumulate in the sub-endothelial space. Here, they can interact with the extracellular matrix, composed mostly of proteoglycans, and induce an inflammatory response primarily mediated by macrophages and T-lymphocytes, starting the process which leads to the development of atherosclerosis [7,8]. There is therefore a role in determining the clinical manifestation of vascular damage caused not only by LDL, but also other atherogenic particles such as very low-density lipoproteins (VLDL), intermediate-density lipoproteins (IDL), remnants, and small dense lipoproteins [9]. Other lipid factors, above those present in metabolic syndrome, are associated with carotid atherosclerosis in post-menopausal women [10]. In a recent study, our research group demonstrated the association between cholesterol contained in VLDL and the presence of carotid plaques [11]. However, the determination of specific lipoprotein subfractions requires sophisticated equipment, which is often used in research but is not used in clinical practice. We therefore questioned whether lipid parameters that are routinely (and easily) investigated could help in evaluating the association with atherosclerosis studied with carotid artery ultrasound. Among the various atherogenic lipid indices proposed, the non-HDL-C/HDL-C ratio seemed most reliable since non-HDL-C (which is the mathematical difference between total cholesterol and HDL-C) represents all atherogenic cholesterols bound to LDL, Lp(a), IDL, or VLDL and to remnants, but this value is corrected for HDL-C, which is a protective factor and therefore appears as the denominator of the fraction. The most studied parameter to investigate atherosclerosis non-invasively is the carotid intima–media thickness (IMT), which is evaluated with carotid ultrasound [12,13]. Many studies have used carotid IMT as a marker for subclinical atherosclerosis and recent meta-analyses have documented a positive association between progression of IMT and cardiovascular risk [14,15,16].

The present study aimed at evaluating the association between the non-HDL-C/HDL-C ratio and subclinical carotid atherosclerosis in a population of post-menopausal women in primary prevention and at verifying the relationship between lipid parameters and the concentration of VLDL-C measured by Lipoprint^®^, the lipid subfraction most strongly correlated to carotid plaques [11]. 

## 2. Materials and Methods

This cross-sectional study involved 220 women who participated in the ATENA Project, which is an epidemiological study conducted in Southern Italy with the objective of studying the environmental, biological, and genetic aetiology of major chronic diseases in women [17]. There were 5062 healthy women who participated in the first round of the ATENA study, partly selected randomly from telephone lists in Naples and partly through responses to health information campaigns, and participants were aged between 30 and 69 years old. The study was approved by the Ethics Committee at University Federico II di Napoli and informed written consent was obtained. During the first visit (10 women each day) the older three subjects—those at potential higher risk of atherosclerotic cardiovascular disease—were invited to undergo carotid arteries ultrasound and additional biochemical tests. A total of 400 women were invited and 310 accepted these additional investigations. Ten years after the first visit, these women were all menopausal and were asked to be followed-up. Two-hundred twenty-eight women accepted undergoing clinical examination and biochemical analysis. Since HDL-C was not available in eight participants, the sample size consisted of 220 women. BMI and waist circumference were measured as indicators of overweight or obesity. A questionnaire was given to all participants for other information, including dietary, social, and smoking history. Brachial systolic and diastolic blood pressure was measured after 5 min of rest using a random zero sphygmomanometer. A second blood pressure was obtained 2 min after the first and an average of the two was used in the study. 11.5% of patients in this group were taking lipid lowering drugs: 11% were taking statins and 0.5% fibrates. Venous blood samples were obtained in the fasting state, between 08.00 and 10.00 in the morning, and investigations were completed on fresh blood (serum), whereas determination of the subfractions of cholesterol (mean LDL size and small LLD score) were carried out with the Lipoprint^®^ system (Quantimetrix Inc, Redondo Beach, CA, USA) on serum samples frozen at −80 °C, within 5 years of obtaining the sample [18]. Concentrations of total cholesterol and triglycerides were determined with standard enzymatic methods. HDL-C was measured after precipitation of VLDL and LDL with phosphotungstic acid. LDL was calculated using the Friedewald formula [19]. Apo-B and Hs-CRP were measured with turbidimetric analysis (Cobas-Mira, Roche, Italy). Blood glucose was measured with the peroxidase method. Insulinaemia was determined by immune-enzymatic method (Ultrasensitive Insulin Elisa; Mercodia, Uppsala, Sweden). Lipoprotein a [Lp(a)] was measured by an ELISA, solid phase two-site enzyme immunoassay, using polyclonal antibodies raised against purified Lp(a) (Mercodia Diagnostics, Uppsala, Sweden).

The insulin resistance index (homeostatic model assessment, HOMA-IR) was calculated using the equation of Matthews et al. [20]. Ultrasonographic study was conducted with high resolution ultrasound by a sonographer certified to conduct echographic examination of atherosclerosis by the Division of Vascular Ultrasound Research, Wake Forest University, Bowman Gray School of Medicine, Winston-Salem, NC, USA, and followed a rigid examination protocol. The segment of the carotid taken for the examination was the 1 cm distal to the common carotid, before the carotid bulb, on both sides. The protocol involved a longitudinal scan of the carotids, with measurements of both the near and far walls, evaluated in anterior, posterior, and lateral projections, using a circumferential approach. The main aim of this examination was to clearly highlight the IMT, on both the near and far walls, in a way that the maximum thickness of the IMT (IMT max) could be selected. Unlike other measures of IMT (IMT mean and IMT mean max), IMT max better reflects the severity, rather than the extent, of atherosclerosis on the carotid wall. Furthermore, an IMT max value > 1.2 mm (average of the IMT max values) together with protrusion of 50% with respect to the neighboring wall, was deemed carotid plaque. Previous data on reproducibility show the above protocol to have a reproducibility coefficient of 0.85 [10].

### Statistical Analysis

Statistical analysis was performed using SPSS, version 19 (SPSS Inc., Chicago, IL, USA). Continuous variables were described as mean and standard deviation. When distribution was not normal, and skewness was >2, variables were transformed into their natural logarithm. Ordinal variables were given as frequencies or percentages. Univariate linear regression was used to evaluate the association between IMT max (expressed as a continuous variable) and the non-HDL-C/HDL-C ratio (which was also expressed as a continuous variable). Multivariate linear regression was used to eliminate the effect of confounding factors. Binary logistic regression was used to test the association between tertiles of non-HDL-C/HDL-C and the presence of carotid plaques, where the risk of plaques was expressed as an odds ratio (OR) with a 95% confidence interval (95% CIs). A multivariate analysis was subsequently completed, with non-lipid coronary risk factors being added as covariates. 

## 3. Results

**Figure 1:**
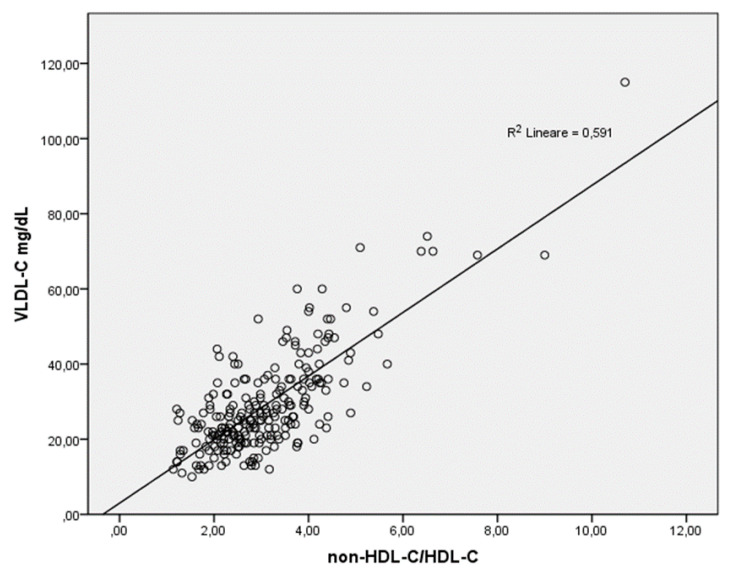
Correlation between non-HDL-C/HDL-C and VLDL-C.

Anthropometric and biochemical characteristics are shown in Table 1, which also reports separate data for women with and without carotid plaques. This study involved 220 women, with a mean age of 63.1 ± 8.2 years, in which 45% had plaques on carotid ultrasound. Most participants were overweight or obese (42% overweight and 29% obese), 35% of women currently smoked, 20% were ex-smokers, and 45% had never smoked; in women who had carotid plaques, these numbers were 36%, 20% and 44%, respectively whereas in the non-plaque group they were 33%, 20% and 47% respectively. These differences were not statistically significant. Participants with carotid plaques, following Student’s t-test, were found to have a higher systolic blood pressure and higher levels of total cholesterol, non-HDL cholesterol, LDL cholesterol, apo-B, triglycerides, and a greater non-HDL-C/HDL-C ratio than those without carotid plaques. After bivariate correlation, the following lipid variables were shown to be significantly positively correlated to the continuous variable of IMT-max: Total Cholesterol: Pearson’s r = 0.20, *p* = 0.003; LDL-C: r = 0.20, *p* = 0.004; non-HDL-C: r = 0.21, *p* < 0.001; non-HDL-C/HDL-C: r = 0.21, *p* = 0.001; LnTG: r = 0.25, *p* < 0.001. HDL-C showed a negative correlation that was not statistically significant (r = −0.13, *p* = 0.062). After adjusting for main non-lipid cardiovascular risk factors (age, systolic blood pressure, smoking, body mass index, Homeostasis model assessment: insulin resistance and high-sensitivity C-Reactive-Protein), multivariate linear regression analysis (Table 2) showed that non-HDL-C/HDL-C ratio was associated to IMT-max The non-HDL-C/HDL-C ratio significantly correlated to VLDL-C measured by Lipoprint^®^ (r = 0.76 and *p* < 0.001) (Figure 1). Finally, to test the association between the presence of carotid plaques on ultrasound and the highest values of lipid parameters routinely obtained, a multivariate logistic regression analysis was conducted in which the presence of carotid plaques was the dependent variable and tertiles of lipid levels represented the categorized independent variables: covariates were inserted as age, BMI, smoking habit, systolic blood pressure, HOMA-IR e Hs-CRP (Table 3). Data showed that higher tertiles of non-HDL and the non-HDL-C/HDL-C ratio, were significantly associated with the presence of carotid plaques (OR 2.34 95%CI 1.05–5.22, *p* = 0.038 and OR 3.47 95%CI 1.51–8.00, *p* = 0.003, respectively), whereas the highest tertile of HDL ensured some protection against the formation of carotid plaques (OR 0.36 95%CI 0.16–0.83, *p* = 0.017) (Table 3).

## 4. Discussion

This study aimed at identifying whether lipid parameters present in routine investigations are associated with carotid plaques in post-menopausal women, and at verifying if these parameters were associated to VLDL-C levels, the lipid subfraction most strongly correlated to carotid IMT. The main findings suggest that the non-HDL-C/HDL-C ratio, which is easily and routinely calculated, is associated with increased carotid intima–media thickness more than other lipid parameters; those higher values of the non-HDL-C/HDL-C ratio confer an increased risk of carotid plaques.

In the Fenofibrate Intervention and Event Lowering in Diabetes (FIELD) study, non-HDL-C/HDL-C ratio was shown to perform better than LDL concentration in risk prediction for both fatal and non-fatal cardiovascular disease [21]. Similarly, in the UK Prospective Diabetes Study (UKPDS), the non-HDL-C/HDL-C ratio had a better performance than non-HDL alone as a predictor of cardiac disease in type 2 diabetes [22]. In addition, a Swedish study in 2014 showed that low non-HDL-C/HDL-C ratio was a better marker of risk for cardiac ischaemia than LDL-C [23]. Finally, it has been recently reported that high non-HDL-C/HDL-C ratio values are associated with an increased risk of coronary artery disease [24].

In post-menopausal women, both the distribution and the characteristics of lipid profile (high levels of total cholesterol, LDL-C, small dense LDL, non-HDL-C/HDL-C ratio are associated to the post-menopausal state) have been previously reported [25].

Anagnostis et al. [26] reported that the ratio between Total Cholesterol/HDL-C increases after menopause. A prospective Chinese study has demonstrated that the numbers of people with higher concentrations of cholesterolaemia, triglyceridaemia, and the Total Cholesterol/HDL-C ratio, increase during menopause with respect to the pre-menopausal phase [27]. 

The non-HDL-C/HDL-C ratio significantly correlates with metabolic syndrome. In a study from Zhao et al. [28], non-HDL-C/HDL-C ratio has been demonstrated to be superior to traditional lipid parameters for assessing the degree of atherosclerosis. In a previous study, non-HDL-C/HDL-C ratio was associated to carotid atherosclerosis in post-menopausal middle-aged women [29]. However, in the present study, we analysed the association of glycaemic indexes and second level lipid metabolism (insulinaemia, HOMA-IR, Lp(a), small dense lipoproteins, Lipoprotein subfractions) to carotid atherosclerosis. 

Small dense LDL-C is associated with subclinical atherosclerosis and Lp(a) contributes to carotid atherosclerosis in menopausal women without metabolic syndrome [10,18]. In the present study, the association between routinely measured lipids and lipid subfractions has been evaluated. Notably, in a previous study, VLDL-C, measured with Lipoprint^®^, was the lipoprotein subfraction that was best associated with subclinical atherosclerosis [11]. Interestingly, in this cohort of postmenopausal women, we found that the non-HDL-C/HDL-C ratio was highly correlated to VLDL-C, suggesting the potential for adopting non-HDL-C/HDL-C ratio as a surrogate marker and potential predictor of early atherosclerosis.

Taken together, the above-mentioned studies demonstrate that many lipid parameters are associated with subclinical atherosclerosis (small dense LDL, Lp(a), VLDL-C) but the non-HDL-C/HDL-C, which is strictly correlated to VLDL-C, showed the best correlation with carotid atherosclerosis, at least in a cohort of menopausal women.

Some limitations should be acknowledged. The cross-sectional nature of this study does not allow us to ascertain the cause–effect relationship between lipid profile and carotid atherosclerosis together with the inability, to verify the incidence of a disease over time. In addition, the rather long intermission (10 years after the first visit) of the ongoing nature of the ATENA project and over 30% lost to follow-up could be considered a potential selection bias. For these reasons, we could not use strong end points (mortality, myocardial infarction, stroke) that may only be available in a prospective study, and were therefore forced to use surrogate end points such as subclinical atherosclerosis studied using ultrasound. However, this method represents an excellent proxy for generalized atherosclerosis [30,31]. Moreover, another limitation of the study is the use of the Friedewald formula for the calculation of LDL-C instead of a direct measurement of LDL-C. The use of Friedewald formula could lead to underestimation of LDL-C, especially in patients with low LDL-C concentration (<100 mg/dL) and/or high triglycerides levels (>200 mg/dL). Conversely, strengths of the study include the use of a solid and validated ultrasound protocol for studying atherosclerosis [32] and the availability of biochemical parameters of lipid and glycaemic metabolism not routinely measured (i.e., apo-B, lipid subfractions, insulin) [31].

In conclusion, although guidelines indicated LDL cholesterol as a primary treatment target for primary prevention of cardiovascular disease and assign non-HDL cholesterol a secondary role as a target in the reduction of risk in patients with elevated triglycerides, this study has demonstrated that, at least in post-menopausal women in primary prevention, the non-HDL-C/HDL-C ratio best correlates with carotid atherosclerosis. Non-HDL-C/HDL-C ratio significantly correlates to VLDL-C, which has shown to be the lipoprotein component most strongly associated to subclinical atherosclerosis. The non-HDL-C/HDL-C ratio could represent the most powerful lipid marker in predicting carotid atherosclerosis since the non-HDL-C/HDL-C ratio combines both the contribution from ‘atherogenic’ components, which are given in the numerator (which we must remember is not only LDL but also VLDL, IDL, small dense lipoproteins, Lp(a) and remnants), and the ‘protective’ components (HDL) which are given in the denominator.

## Figures and Tables

**Table 1:** Baseline characteristics of the population and characteristics of the groups with and without carotid plaques.

Variables	Whole Cohort	Women without Carotid Plaques	Women with Carotid Plaques	*p*
	*n* = 220	*n* = 120	*n* = 100	
Age (years)	63.1 ± 8.2	60.8 ± 8.1	66.1 ± 7.2	**<0.001**
SBP (mmHg)	143.1 ± 21.2	138.7 ± 20.2	149.0 ± 21.5	**<0.001**
DBP (mmHg)	81.5 ± 8.9	81.0 ± 9.3	82.0 ± 8.4	0.40
BMI (Kg/m^2^)	28.1 ± 4.6	27.9 ± 4.6	28.6 ± 4.7	0.31
WC (cm)	91.4 ± 11.1	90.6 ± 11.0	93.0 ± 11.4	0.12
Glucose (mg/dL)	105.6 ± 25.0	103.4 ± 17.9	108.8 ± 31.8	0.12
HOMA-IR	1.8 ± 1.3	1.7 ± 1.2	1.9 ± 1.4	0.20
Ln_HOMA-IR	0.38 ± 0.63	0.36 ± 0.57	0.42 ± 0.69	0.47
Chol (mg/dL)	224.5 ± 38.4	216.8 ± 38.2	233.4 ± 37.4	**0.001**
HDL (mg/dL)	57.9 ± 13.6	58.4 ± 13.4	56.6 ± 13.5	0.32
Non-HDL (mg/dL)	166.7 ± 39.7	158.4 ± 38.0	176.8 ± 39.6	**0.001**
non-HDL/HDL (mg/dL)	3.1 ± 1.2	2.9 ± 1.2	3.3 ± 1.2	**0.009**
LDL (mg/dL)	144.5 ± 34.4	137.8 ± 33.0	152.3 ± 34.4	**0.002**
TG (mg/dL)	110.9 ± 56.7	102.9 ± 52.7	122.4 ± 60.9	**0.012**
Ln_TG	4.6 ± 0.4	4.5 ± 0.4	4.7 ± 0.4	**0.001**
Apo-B (mg/dL)	1.11 ± 0.22	1.06 ± 0.22	1.16 ± 0.21	**0.001**
Mean LDL size (nm) ^§^	27.6 ± 3.5	27.1 ± 3.3	27.0 ± 3.3	**0.049**
LDL score (% small dense LDL) ^§^	3.69 ± 6.1	3.35 ± 5.7	3.93 ± 6.3	0.289
VLDL-C (mg/dL) ^§^	29.1 ± 13.7	27.0 ± 12.0	31.7 ± 15.0	**0.01**
Lp(a) (mg/dL)	23.9 ± 26.9	21.9 ± 26.1	25.4 ± 26.9	0.330
CRP (mg/L)	2.68 ± 4.07	2.68 ± 4.13	2.65 ± 4.08	0.970
Ln_CRP	0.41 ± 1.09	0.36 ± 1.14	0.45 ± 1.01	0.521
CC IMT-max (mm)	1.26 ± 0.38	1.05 ± 0.11	1.52 ± 0.43	**<0.001**

Mean ± SD; Age (years); SBP: Systolic Blood Pressure (mmHg); Diastolic Blood Pressure (mmHg); BMI: Body Mass Index (kg/m^2^); WC: Waist Circumference (cm); Glucose (mg/dL); HOMA-IR: Homeostasis model assessment: insulin resistance; Ln_HOMA-IR (HOMA-IR logarithmically transformed); Chol: Total Cholesterol (mg/dL); HDL: HDL-Cholesterol (mg/dL); Non-HDL: Non-HDL-Cholesterol (mg/dL); Non-HDL/HDL: non-HDL-Cholesterol/HDL-Cholesterol (ratio); LDL: LDL-Cholesterol (mg/dL); TG: Triglycerides (mg/dL); Ln_TG (TG logarithmically transformed); Apo-B (g/L); Mean LDL size (nm); LDL score (% small dense LDL); VLDL-C: Very low density lipoprotein cholesterol (mg/dL); Lp(a): Lipoprotein(a) (mg/dL); CRP: High-sensitivity C-Reactive-Protein (mg/L); Ln_CRP (CRP logarithmically transformed); CC IMT-max:Common Carotid maximum thickness (mm).^§^ Determination of VLDL-C concentrations and mean LDL size and score was performed on frozen serum samples by using electrophoresis Lipoprint^®^ System (Quantimetrix Inc., Redondo Beach, CA, USA). Bolded number means *p* < 0.05.

**Table 2:** Relationship between lipid parameters and carotid IMT.

Variable	β	95% CI	*p*
Total Chol (mg/dL) *	0.001	0.000 to 0.002	0.13
LDL (mg/dL) *	0.001	0.000 to 0.003	0.073
Ln-TG *	0.109	−0.001 to 0.220	0.052
Non-HDL/HDL (mg/dL) *	0.039	0.001 to 0.076	**0.042**

Carotid IMT: Common Carotid maximum thickness (mm); Total Chol: Total Cholesterol (mg/dL); LDL: LDL-Cholesterol (mg/dL); Non-HDL: Non-HDL-Cholesterol (mg/dL); HDL: HDL-Cholesterol (mg/dL). Non-HDL/HDL: non-HDL-Cholesterol/HDL-Cholesterol (ratio); Ln-TG: Triglycerides (Logarithmically transformed). * Adjusted for age, systolic blood pressure (SBP), smoking, body mass index (BMI), Homeostasis model assessment: insulin resistance (HOMA-IR) and high-sensitivity C-Reactive-Protein (Hs-CRP). Bolded number means *p* < 0.05.

**Table 3:** Association between tertiles of lipid parameters and carotid plaques.

	II Tertile vs. I Tertile (Reference)			III Tertile vs I Tertile (Reference)		
Variable	OR	95% Confidence Intervals	*p*	OR	95% Confidence Intervals	*p*
Chol (mg/dL) *	1.89	0.83–4.28	0.12	1.96	0.89–4.35	0.096
LDL (mg/dL) *	1.24	0.55–2.80	0.60	2.19	0.98–4.85	0.054
Non-HDL (mg/dL) *	1.17	0.52–2.59	0.70	2.34	1.05–5.22	0.038
HDL (mg/dL) *	0.47	0.20–1.09	0.08	0.36	0.16–0.83	0.017
Non-HDL/HDL (mg/dL) *	1.59	0.71–3.57	0.26	3.47	1.51–8.00	0.003
Ln-TG *	1.59	0.72–3.48	0.25	2.18	0.94–5.08	0.071

Chol: Total Cholesterol (mg/dL); LDL: LDL-Cholesterol (mg/dL); Non-HDL: Non-HDL-Cholesterol (mg/dL); HDL: HDL-Cholesterol (mg/dL); Non-HDL/HDL: non-HDL-Cholesterol/HDL-Cholesterol (ratio); Ln-TG: Triglycerides (Logarithmically transformed). * Adjusted for age, systolic blood pressure (SBP), smoking, body mass index (BMI), Homeostasis model assessment: insulin resistance (HOMA-IR) and high-sensitivity C-Reactive-Protein (Hs-CRP).

## Data Availability

Not applicable.
